# Battlefronts of evolutionary conflict between bacteria and animal hosts

**DOI:** 10.1371/journal.ppat.1008797

**Published:** 2020-09-17

**Authors:** Omoshola Aleru, Matthew F. Barber

**Affiliations:** 1 Institute of Ecology & Evolution, University of Oregon, Eugene, Oregon, United States of America; 2 Department of Biology, University of Oregon, Eugene, Oregon, United States of America; Duke University School of Medicine, UNITED STATES

## What is evolutionary conflict?

**Fig 1:**
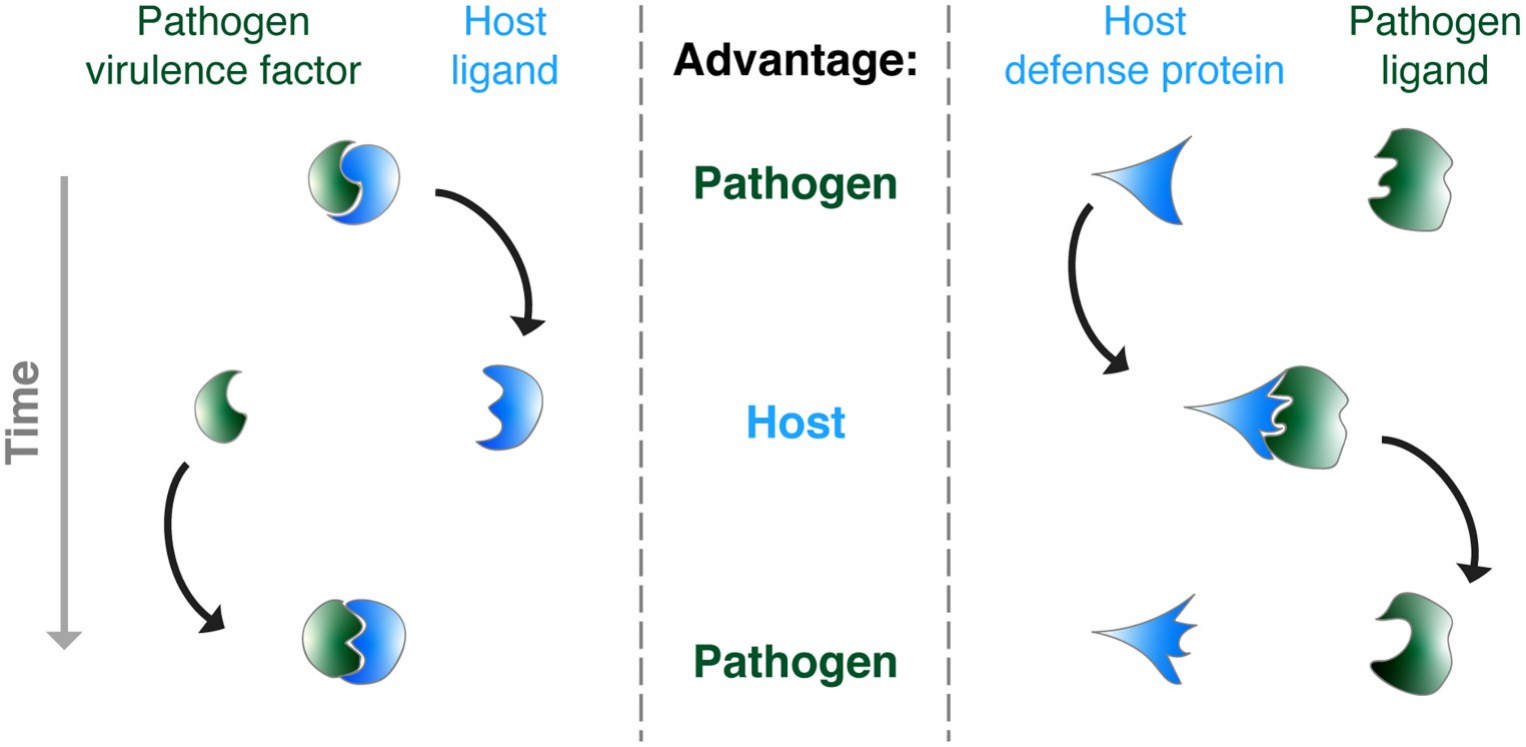
Conceptual overview of host–pathogen evolutionary conflicts. Natural selection in microbial pathogens (green) is predicted to favor mutations that either enhance the activity of virulence factor proteins (left panel) or avoid detection by host immune defenses (right panel). Conversely, mutations in host proteins (blue) that counteract virulence factors or enhance detection of microbial ligands are also predicted to quickly adapt due to positive selection. These antagonistic molecular interactions can give rise to evolutionary conflicts in which neither host nor pathogen ever gains a permanent advantage. Black arrows represent the continual processes of mutation and selection that fuel evolutionary conflicts.

Conflict is pervasive in nature, as observed in the struggle between predators and prey, competition for mates, as well as between pathogenic microbes and their hosts. The burden imposed by pathogens can place strong selective pressure on host populations to evolve resistance to infection [1]. Conversely, host immune responses promote the repeated evolution of defensive countermeasures by microbial pathogens. This antagonism can give rise to evolutionary conflicts, including “Red Queen” dynamics, in which pathogens and hosts are forced to continually adapt to maximize their relative fitness (Fig 1) [2,3]. Consistent with the existence of such conflicts, immune system components have been shown to be among the most rapidly evolving genes in animal genomes [4–7]. These observations can reflect the rapid spread of new beneficial mutations in populations over time, a process termed positive selection. Unique genetic signatures are used to infer positive selection between and within species, including elevated rates of nonsynonymous nucleotide substitutions relative to synonymous substitutions in protein-coding genes (also termed dN/dS or ω), as well as to measure the loss of genetic variation around a locus associated with a recent selective sweep. Genomic studies further support the long-held theory that host–pathogen interactions are major drivers of natural selection and adaptation across diverse taxa [4,6–8].

The past 15 years have seen a powerful integration of genetic and experimental approaches to identify instances of host–pathogen conflict as well as empirically test how conflicts shape immunity and disease [9]. Such approaches have pinpointed new molecular functions underlying host defense [10,11], identified completely new genes or pathways involved in disease susceptibility [12,13], and revealed new determinants of pathogen tropism [14–16]. Host–pathogen evolutionary conflicts thus provide powerful systems for dissecting mechanisms of infectious disease pathogenesis.

## How have host immune defenses been shaped by evolutionary conflicts with bacteria?

**Fig 2:**
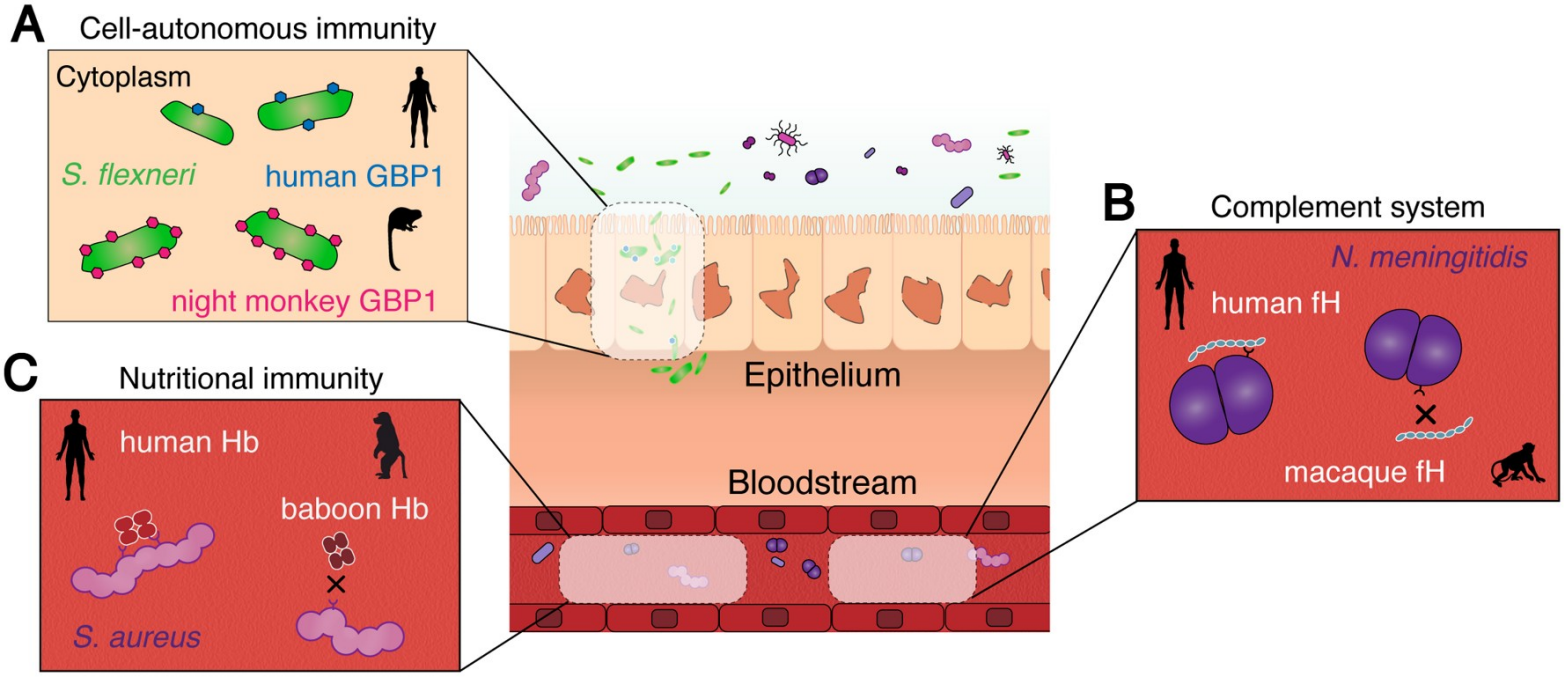
Interfaces of evolutionary conflict between animals and pathogenic bacteria. Three representative examples of conflicts involving human bacterial pathogens are illustrated. **(A)** Human GBP1 recognizes *Shigella flexneri* via a C-terminal PBM. Diversification within the PBM of GBP1 in night monkeys and other New World primates confers enhanced recognition to *S*. *flexneri* relative to human GBP1. **(B)** Pathogenic *Neisseria* avoid detection by the complement alternative pathway through recruitment of fH to their surface. Human fH exhibits the strongest binding to *Neisseria meningitidis*, whereas nonhuman primate fH is poorly recognized by these bacteria. Regions of fH corresponding to bacterial recognition sites are known to exhibit strong signatures of repeated positive selection. **(C)** *Staphylococcus aureus* scavenges host heme using the IsdB receptor. Rapidly diverging sites within alpha and beta hemoglobin in primates impair recognition by IsdB, contributing to host nutritional immunity. fH, factor H; GBP1, guanylate binding protein 1; IsdB, iron-regulated surface determinant B; PBM, polybasic motif.

An established and growing body of work has characterized instances of evolutionary conflict between animals and viruses [5,9]. More recently, studies have begun to emerge revealing molecular details of conflicts driven by cellular pathogens including bacteria, fungi, and parasites. Below, we highlight recent advances in our understanding of evolutionary conflicts between animal hosts and pathogenic bacteria (Fig 2), as well as discuss future areas of study in this burgeoning field.

### Cell-autonomous immunity

Many metazoan cell types possess the intrinsic ability to detect and defend against pathogens, a feature termed cell-autonomous immunity [17]. Cell-autonomous immunity represents an ancient arm of host defense which can be triggered by cytosolic pattern recognition receptors as well as extracellular signaling molecules such as interferon [18]. Evidence is accumulating that intracellular bacteria have instigated repeated evolutionary conflicts with several critical cytosolic host defense factors. Key among these are inflammasomes, protein complexes that form in response to detection of various intracellular pathogens [19,20]. Assembly of distinct inflammasomes containing inflammatory caspases as well as nucleotide-binding oligomerization domain-like receptor (NLR) proteins can ultimately lead to inflammatory host cell death, termed pyroptosis, along with the release of proinflammatory cytokines. Recent studies have illustrated that several mammalian NLR family proteins have undergone rapid divergence in domains responsible for bacterial pathogen sensing. In one case, mapping the flagellin-binding region of the neuronal apoptosis inhibitory proteins (NAIPs), a subgroup of mouse NLRs, simultaneously revealed that this domain exhibits high dN/dS across the rodent lineage, suggestive of repeated positive selection [21]. Given the diversity of flagellin proteins found in bacteria and the expansion of NAIP gene copy number in rodents, these data support a model in which NAIPs have undergone repeated adaptation to recognize an array of bacterial flagellins encountered during infection. More recently, Chavarría-Smith and colleagues provided evidence that primate NLRP1 is rapidly diverging within an N-terminal region that undergoes bacterial-mediated proteolytic cleavage leading to inflammasome activation [22]. In light of additional studies that collectively revealed how NLRP1 N-terminal degradation functions as a sensor of bacterial-induced proteolysis [23,24], signatures of natural selection in NLRP1 family proteins suggest that genetic variation in this region can enhance surveillance against a range of intracellular bacterial effectors.

An additional group of host factors that contribute to defense against intracellular pathogens are interferon-stimulated GTPases, including the guanylate binding proteins (GBPs). Molecular, cellular, and genetic studies have demonstrated that GBPs serve numerous roles in cell-autonomous immunity, from recognizing pathogen-containing cellular compartments to promoting inflammasome activation and directly binding to microbial cell surfaces in the cytosol [25–27]. Mammals encode a variable number of GBP paralogs, with humans possessing seven, as well as several pseudogenes. Oligomerization on a target surface allows GBPs to cooperatively bind to pathogens or pathogen-containing membranes as well as recruit additional defense factors to limit microbial replication. A series of recent studies has illustrated mechanisms by which human GBP1 recognizes cytosolic Gram-negative bacteria [28–30]. Detection of pathogenic *Shigella* by human GBP1 relies on the presence of a C-terminal prenylation motif adjacent to a stretch of basic amino acids, termed the polybasic motif (PBM). The PBM contributes to direct recognition of the Gram-negative bacterial envelope, allowing GBP1 to serve as an oligomeric lipopolysaccharide sensor [31–33]. We and our collaborators recently demonstrated that this PBM displays signatures of recurrent positive selection in primate GBP1 and GBP2, suggestive of adaptation in response to conflicts with cytosolic microbes (Fig 2A) [34]. Swapping the PBM between primate GBP1 proteins revealed that several New World monkeys possess enhanced bacterial-targeting activity relative to humans, illustrating how beneficial mutations are capable of augmenting GBP defensive functions. Although much more remains to be uncovered regarding the evolution of cell-autonomous immunity, these recent studies demonstrate how selective pressures imposed by bacterial pathogens can rapidly shape the activity of host defense factors.

### The complement system

The complement system comprises a large network of soluble and cell-surface proteins in animals that recognize nonself molecular features, facilitating the direct rupture of microbial membranes as well as stimulating leukocyte recruitment and inflammation [35]. Successful pathogens in turn have evolved a variety of mechanisms to avoid or down-regulate complement activation. One effective means of pathogen complement evasion involves the recruitment of regulatory proteins, which normally serve to protect host cells from inappropriate complement activation. By recruiting these host regulators, microbes can effectively “cloak” themselves against this potent defense system. Although core components of the complement system are widely conserved in animals, genome-wide studies have also revealed that numerous complement genes exhibit evidence of repeated positive selection between species. A recent study highlighted such signatures in several complement regulators, including factor H (fH) and C4 binding protein A (C4BPA) [36]. fH is a major soluble host complement regulator that prevents activation of the alternative complement pathway, while C4BPA prevents activation of the classical and lectin pathways. Moreover, rapidly evolving domains in both fH and C4BPA map to known binding sites of diverse pathogenic bacteria [37,38]. These findings strongly suggest that antagonism by bacteria has prompted evolutionary conflicts with host complement regulators over millions of years of animal evolution (Fig 2B). Given that humans also encode native fH-like proteins that are hypothesized to serve as mimics against pathogen hijacking [39], future work aimed at delineating the relationship between host and microbial variation at this interface could reveal important determinants of pathogen complement evasion, as well as potential strategies to counteract this process.

### Nutritional immunity

In addition to evading dedicated host immune defenses, bacteria and other cellular pathogens must acquire nutrients to survive and grow during an infection. Nutrient metals are particularly scarce and must be scavenged by bacterial pathogens within the host. The active sequestration of metals such as iron, manganese, and zinc provides an important host defense mechanism termed nutritional immunity [40–42]. We and others have shown that multiple components of host nutritional immunity exhibit genetic signatures of evolutionary conflicts similar to more canonical immune defense factors mentioned above. Previous work illustrated that transferrin, the principle bloodstream iron transport protein in vertebrates, has undergone repeated positive selection specifically at the interface with the Gram-negative bacterial receptor transferrin binding protein A (TbpA) [43]. This outer-membrane protein facilitates the acquisition and transport of iron into bacterial cells from transferrin, making it a major virulence factor in several human pathogens, including *Neisseria gonorrhoeae*, *N*. *meningitidis*, *Haemophilus influenzae*, and *Moraxella catarrhalis* [44]. Although evidence of repeated adaptation was also observed in the transferrin paralog lactoferrin, rapidly evolving regions of this protein suggest that selection has acted primarily on new antimicrobial functions that have emerged since its divergence from transferrin, rather than iron binding [45]. More recently, we and others discovered that mammalian heme binding proteins are also rapidly evolving at molecular interfaces recognized by bacterial pathogens [46,47], most notably the hemoglobin alpha and beta subunits targeted by diverse bacterial and eukaryotic pathogens. Mutating positions subject to positive selection in both transferrin and hemoglobin are sufficient to impair recognition by pathogenic bacteria, supporting the hypothesis that variation in these host factors could provide a benefit to host fitness during bacterial infections (Fig 2C) [46]. It remains an open question as to how competition for other nutrients may lead to evolutionary conflicts between bacteria and animal hosts.

## What are emerging questions regarding bacterial–host evolutionary conflicts?

The studies highlighted here illustrate that evolutionary conflicts between bacteria and animal hosts are widespread and have important consequences for infectious disease pathogenesis and immunity. However, many unanswered questions remain. Beyond immune evasion and nutrient acquisition, bacteria encode a wide variety of virulence factors that could also give rise to conflicts. Recent work has provided evidence of repeated adaptation among host proteins targeted by bacterial toxins [48,49], consistent with this hypothesis. Given that many bacterial virulence factors are also encoded by related commensal microbes, pathogen-driven conflicts could also hold the potential to restrict the host species tropism of commensal members of the microbiota. In this case, we would expect that commensal bacteria are also forced to adapt in response to rapidly evolving host factors required for colonization, even when they themselves are not the driving agents of conflict. Since the effectiveness of host barrier defenses can depend on the presence of the microbiota [50–53], it will be important to test how evolution of commensal microbes can in turn shift the balance of host–pathogen conflicts. For example, host genetic variation in iron-binding proteins may only be effective at excluding pathogens when particular host-adapted commensal microbes are present to out-compete them [54]. We expect the coming years will continue to expand our understanding of how and why bacterial–host evolutionary conflicts arise, as well as provide opportunities to apply this knowledge in humanity’s ongoing battles with microbial pathogens.
